# Suppression of SLC39A6‐CREB1 axis in liver cancer causes PCK1‐mediated mitochondrial dysfunction

**DOI:** 10.1111/cpr.13527

**Published:** 2023-07-12

**Authors:** Ze Yu, Cheng Chen, Hongpeng Gu, Jinliang Dong, Yingjie Zhang, Jie Wang, Haijie Ma, Guoqiang Zhang

**Affiliations:** ^1^ Department of General Surgery, Zhoushan Hospital Wenzhou Medical University Zhoushan Zhejiang China; ^2^ The Laboratory of Cytobiology and Molecular Biology, Zhoushan Hospital Wenzhou Medical University Zhoushan Zhejiang China; ^3^ The Second Affiliated Hospital Zhejiang University School of Medicine Hangzhou Zhejiang China; ^4^ Department of Oncology, Xiangyang Central Hospital Affiliated Hospital of Hubei University of Arts and Science Xiangyang Hubei China; ^5^ Department of General Surgery, Zhoushan Hospital Zhejiang University School of Medicine Zhoushan Zhejiang China

Dear Editor,

Hepatocellular carcinoma is one of the most common cancers worldwide and represents a major global health‐care challenge.^1^ Infection with hepatitis virus, drinking excessive alcohol or suffering from non‐alcoholic fatty liver disease are all known risk factors for liver hepatocellular carcinoma (LIHC).^2^ A complete understanding of the molecular mechanisms underlying LIHC progression and discovering new master regulators and their functional mechanism is of great significance which will contribute to the identification of novel diagnosis and therapeutic targets in LIHC treatment. Zinc, an essential trace element, plays various pivotal roles in numerous biochemical and physiological processes, such as growth, survival and metabolism.^3^ Notably, in liver, zinc is needed for activating many enzymes, such as glutamate dehydrogenase, and superoxide dismutase.^4^, ^5^ Over time, the expanding experimental and clinical evidence had begun to determine the role of zinc in the development of individual cancers.^6^, ^7^ As one of the 14 members of zinc transporter *SLC39A*, *SLC39A6* (*ZIP6*) facilitates the influx of Zinc into the cytosol.^8^ Despite *ZIP6* participates in cancer cell proliferation and migration, little is known about the association of *ZIP6* with the progression of human LIHC. Here, our study suggested a novel ZIP6‐CREB1 axis promoting liver cancer growth, providing mechanistic insights explaining in part how *ZIP6* functions in liver cancer, and *ZIP6*, act as a novel regulator of cancer progression, could be an attractive therapeutic target for LIHC.

The process of bioinformatics analysis in the research is depicted in Supplementary Figure S1. First, an non‐negative matrix factorization cluster analysis was performed including these 14 *SLC39A*‐related genes, and *k* = 2 was determined by the comprehensive correlation coefficient. The Cancer Genome Atlas‐Liver hepatocellular carcinoma (TCGA‐LIHC) samples were then divided into two different clusters (Cluster 1 and Cluster 2). Consensus matrix heat maps with *k* = 2 showed clear boundaries and minimal interference between subgroups, showing stable clusters in the samples (Figure 1A; Supplementary Figure 2A–G). And we compared the survival rates between two groups with different *SLC39A* statuses, compared to Cluster 2, patients in Cluster 1 had a worse prognosis (Figure 1B). In order to better understand the *SLC39A6* status between the two clusters, the volcano plot of differentially expressed genes (DEGs) were shown in Supplementary Figure 2H, and data showed that these DEGs were significantly related to cell proliferation and metabolic process, such as cell cycle and glycolysis (Supplementary Figure 2I).

**FIGURE 1:**
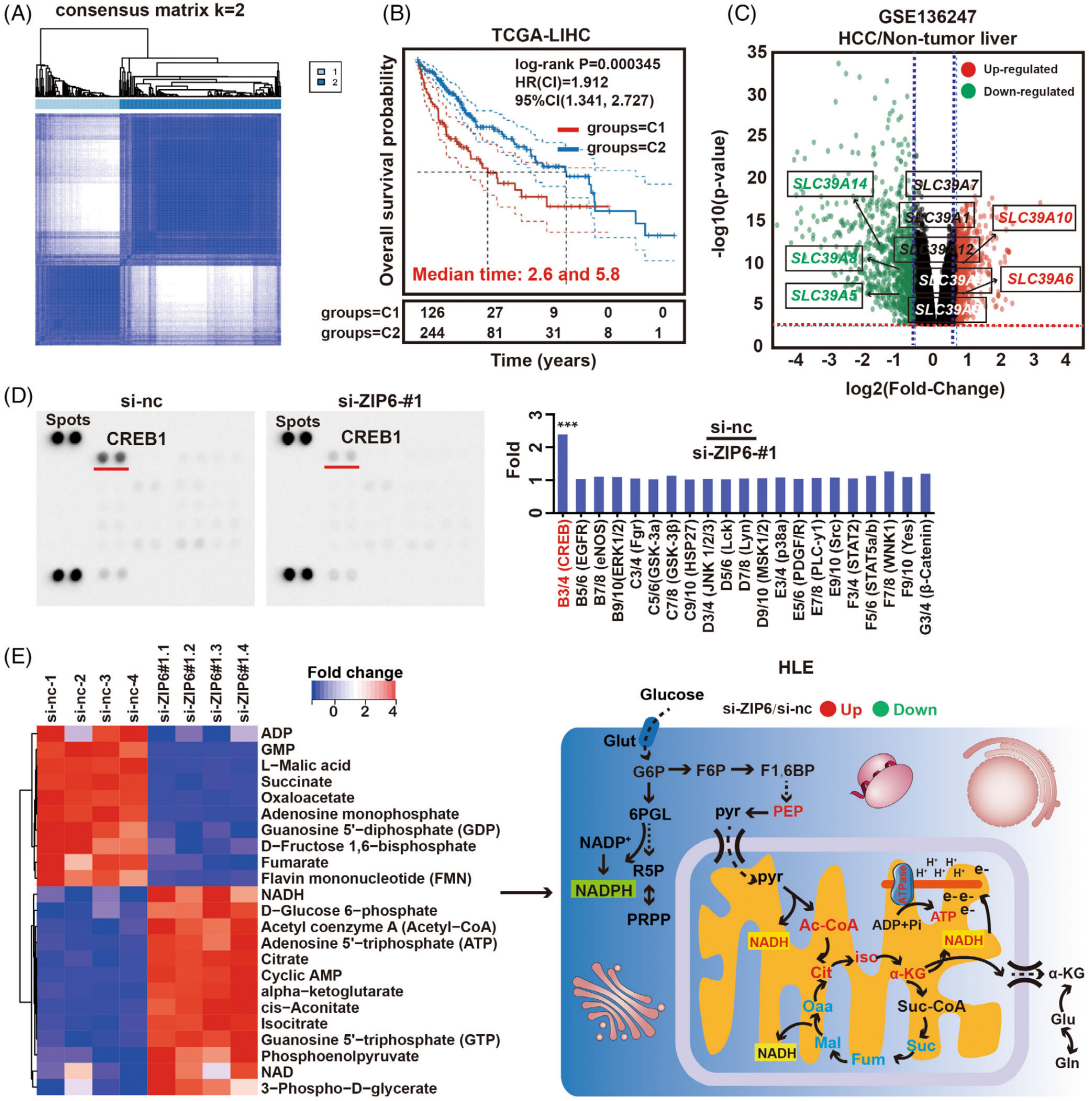
Inhibition of ZIP6‐CREB1 axis restrains cell proliferation. (A) Consensus matrix heat‐map for *k* = 2. (B) Kaplan–Meier survival analysis of LIHC patients in Cluster 1 (*N* = 127) and Cluster 2 (*N* = 244). (C) Volcano plot of mRNA expression for *SLC39A* members in liver cancer versus non‐tumour liver (GSE136247). (D) Images of the phospho‐kinase membrane array. (E) Schematic illustrating the metabolites that are increased (red) or decreased (blue) in SLC39A6 knock‐down HLE cells.

The TCGA dataset was used to obtain RNA‐sequencing expression profiles. Ultimately, 371 samples were selected for analysis. Survival differences between the groups were compared using the log‐rank test. SLC39A2, SLC39A6, SLC39A10, as well as the SLC39A14 (all localized to the cell membrane) risk score was compared using receiver operating characteristic (ROC) analysis. Based on this analysis, we performed LASSO regression to screen for SLC39As that were related to prognosis (Supplementary Figure 3A). Using the 4‐mRNA signature, we calculated the scores, ranked them and classified patients with LIHC patients into high‐ and low‐risk groups by the median value. As the risk score of patients with LIHC increased, the expression levels of risk genes (*SLC39A6* and *SLC39A10*) were obviously upregulated (Supplementary Figure 3B). The Kaplan–Meier survival curve and the log‐rank test both concluded that patients with a high‐risk score died more frequently than those with a low‐risk score (Supplementary Figure 3C). In addition, the time dependent ROC analysis indicated the area under curve for 1‐, 3‐ and 5‐year overall survival (OS) were 0.674, 0.646 and 0.642, respectively (Supplementary Figure 3D). These results confirm the validity of our risk scoring model, and ZIP6 is a prognostic risk factor for LIHC. Subsequently, we obtained the gene expression data from GES136247 and found that *SLC39A6* was upregulated in liver cancer samples (Figure 1C). Furthermore, Hepatocellular carcinoma expression atlas (HCCDB) website and University of ALabama at Birmingham Cancer (UALCAN) webserver revealed *SLC39A6* expression was elevated as the tumour stage and nodal metastasis status progressed (Supplementary Figure 4A,B). Kaplan–Meier analysis and Cox regression model (forest plot) displayed that reduced *SLC39A6* levels were correlated with better OS (Supplementary Figure 4C–E). Combining the findings above, *SLC39A6* can be used as a diagnostic and prognostic biomarker in LIHC patients.

To further investigate the oncogenic role of *SLC39A6* in LIHC, we observe the differentially expressed genes in the two groups, that is *SLC39A6*‐high expression group (50%, *N* = 186 or 25%, *N* = 93) and *SLC39A6*‐low expression group (50%, *N* = 185 or 25%, *N* = 92), respectively, to obtain the clues about the function of *SLC39A6*. KEGG analysis of the differentially expressed genes in the two groups indicated a notable enrichment of cell cycle, pyruvate metabolism and glycolysis pathway, and the results of GO analysis also suggested in the group with low *SLC39A6* expression, the pathways involved in pyruvate metabolism and carboxylic acid catabolic/biosynthetic process were significantly up‐regulated (Supplementary Figure 5A,B). Moreover, results from the correlation analysis revealed that, in addition to tumour inflammation, all of the tumour malignant phenotypes were positively correlated with the expression level of *SLC39A6*. Beyond that, interestingly, the genes linked to reactive oxygen species (ROS) generation, showed a negatively correlation with *ZIP6* (Supplementary Figure 5C). We classified the negatively correlated genes of *SLC39A6* through LinkedOmics database, and discovered that most of these genes were associated with mitochondrial respiratory chain complex (Supplementary Figure 5D,E). This result was consistent with the gene set enrichment analysis result of *ZIP6* (Supplementary Figure 5F).

To investigate the effect of SLC39A6 on cell cycle, we used the spearman analysis to explore the correlation between *SLC39A6* mRNA expression and cell cycle marker genes in LIHC samples, results showed that *SLC396A* was positively correlated with major cell cycle marker genes, which suggests that it has a positive regulatory function on cell proliferation (Supplementary Figure 6A). To further determine the biological function of *SLC39A6*. siRNA was designed and transfected into the cells. Soon afterwards, cell cycle‐related genes were detected by quantitative real‐time PCR (qPCR). Results indicated *SLC39A6* knockdown induced marked downregulation of *CCND1*, *CCNE1*, *CDC6*, *ABL1*, *CCDA2* and *CCNB1* (Supplementary Figure 6B). CCK8 assays revealed knocking‐down SLC39A6 repressed the proliferation of cells (Supplementary Figure 6C). Phosphorylation‐profiling of 22 arrayed kinases in HLE cells transfected with si‐ZIP6 revealed significant p‐CREB reduction, compared with si‐nc group (Figure 1D). These findings suggested CREB1 was a downstream regulatory transcription factor of ZIP6.

Next, targeted metabolomics analysis revealed the HLE cells knocked‐down *ZIP6* contained larger amount of acetyl‐CoA, citrate, isocitrate, α‐ketoglutarate, NADH and ATP. All of them participated in the electron transport chain (ETC). Interestingly, in addition to these metabolites, other intermediates in the TCA cycle, such as succinate, fumarate, malate and oxaloacetate were significantly reduced. Notably, the abundance of phosphoenolpyruvate (PEP) also increased after loss of ZIP6 (Figure 1E). Western blot assays showed the protein levels of COX17 and NDUFS3 were significantly elevated via knocking‐down ZIP6 for 48 h (Figure 2A). Meanwhile, qPCR results showed the key genes of mitochondrial ETC complex were elevated after silencing *ZIP6* (Supplementary Figure 7A). Briefly, these observations suggested that loss of ZIP6 increased mitochondrial respiration, accompanied by a significant increase in the ETC byproduct ROS after 96 h. Similarly, meanwhile, as with our previous envision, mitochondrial depolarization occurred in *SLC39A6* knockdown cells (Figure 2B; Supplementary Figure 7B). Based on these data, we propose that SLC39A6 could suppress the generation of mitochondrial ROS to maintain the survival of tumour cells. Once ZIP6 is knocked down, accumulated ROS in cells significantly disrupts mitochondrial function.

**FIGURE 2:**
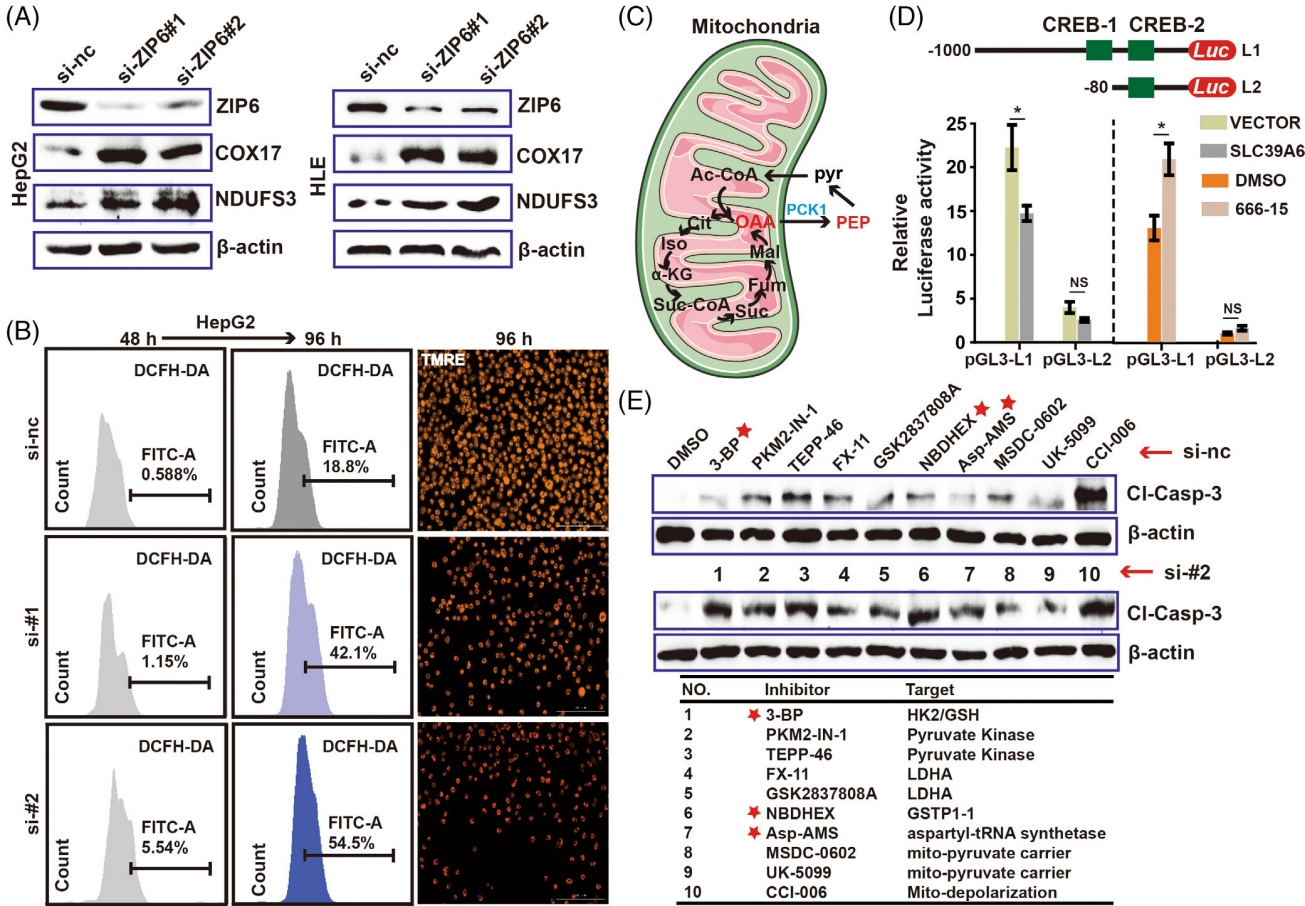
Inhibition of ZIP6‐CREB1‐PCK1 axis causes mitochondrial dysfunction. (A) Protein levels of COX17 and NDUFS3 in SLC39A6 knockdown cells. (B) ROS detection and mitochondrial membrane potential assay for cells treated with si‐SLC39A6 mimics. (C) Schematic diagram for the metabolic function of PCK1. (D) Schematic illustration of luciferase reporters containing different fragments and mutants (deletion) of the PCK1 promoter region. (E) Western blot analysis of Cl‐Caspase‐3 in HLE cells treated with a variety of mitochondrial metabolism inhibitors combined with si‐nc group or knocked‐down SLC39A6.

ZIP6 acts as a zinc transporter to regulate cellular zinc homeostasis. Based on our current study, we can reasonably speculate ZIP6 mediates zinc‐stimulated activation of CREB. Activation of CREB was significantly suppressed in HLE and HepG2 cells treated with si‐*zip6* mimics compared to the control siRNA, and this result was consistent with that of the zinc chelator TPEN group (Supplementary Figure 7C). The endogenous zinc finger transcription factor, ZNF24 and ZNF207, both of them are regulated by intracellular zinc.^9^, ^10^ Depleting the intracellular zinc in both HLE and HepG2 cells with the zinc chelator TPEN significantly reduced the protein levels of ZNF24 and ZNF207, whereas knockdown of ZIP6 did not (Supplementary Figure 7D,E). Given the above, existing findings suggested that CREB activation (phosphorylation) is dependent on the expression levels of ZIP6. University of California Santa Cruz (UCSC) database revealed the mRNA levels of ZIP6 was also positively correlated with that of CREB1, and negatively correlated with genes of NADH family (NDUFA1, NDUFB1 and NDUFS3; Supplementary Figure 7F). Then, we performed visual sample selection and cluster identification for single‐cell sequencing data from GSE3064818. Single‐cell sequencing revealed that the correlations among *SLC39A6*, *CREB1*, *COX17* and *NDUFS3* were consistent with the data from database UCSC (Supplementary Figure 7G). Given that above results, we speculated that the regulation of mitochondrial respiration by SLC39A6 may be related to transcription factor CREB1. Next, GO analysis of the CREB1 negatively correlated genes demonstrated these genes mainly mapped to cytochrome complex, and NADH dehydrogenase complex, which are both important processes in mitochondrial respiration (Supplementary Figure 8A). Among these genes, 52 genes (91.2%) were appeared both in SLC39A6/CREB1‐negative correlation group (Supplementary Figure 8B). Moreover, NADH dehydrogenase complex assembly belong to the biological pathway of SLC39A6/CREB1 overlap negatively correlation (Supplementary Figure 8C), and ROS generation is negatively correlated with CREB1 (Supplementary Figure 8D). Looking at the above factors, the SLC39A6‐CREB1 axis mainly impacts mitochondrial respiration and ROS generation by negatively regulating NADH dehydrogenase complex assembly.

To further determine the regulatory mechanism between mitochondrial respiration and SLC39A6‐CREB1 axis, a Gene‐MANIA network database was then used to explore the targets of CREB1 (Supplementary Figure 8E). After a comprehensive analysis, we found *PCK1* not only show significant negative correlation with *SLC39A6* and *CREB1* (Supplementary Figure 8F), but also is a key enzyme catalysing oxaloacetate to PEP (Figure 2C). It is exciting that this result gave us identical trends as that of the previous metabolomics analysis. Therefore, *PCK1*, the main control point for the regulation of gluconeogenesis, is highly likely to be the downstream effector of SLC39A6‐CREB1 axis in our study. In addition, qPCR was used to quantify the *PCK1* expression after over‐expressing or knocking‐down *SLC39A6* (Supplementary Figure 8G). Inhibitor 666‐15 also significantly increased the *PCK1* mRNA and protein levels (Supplementary Figure 8H). Using online software hTFtarget, we found PCK1 may be regulated by CREB1 (Supplementary Figure 8I). Potential transcription factor CREB1 binding sites in the promoter and 5’‐UTR regions of PCK1 were predicted using the hTFtarget, JASPAR, and TRANSFAC websites. We analysed the 1000 bp DNA sequence upstream and 200 bp DNA sequence downstream of PCK1, found three candidate regions through Venn diagram analysis (Supplementary Figure 8J). Results of the dual‐luciferase activity showed that the luciferase activity of the promoter in 5′‐UTR regions of PCK1 was decreased in the group which overexpressed SLC39A6, compared with the control group, and the luciferase activity increased with the use of 666‐15 (Figure 2D). Moreover, PCK1 transcriptional activity was significantly inhibited overexpressed SLC39A6 when we deleted the sequences of the −90/−83 PCK1 promoter. However, deletion the sequences of +99/+103 PCK1 5′‐UTR would promote the transcriptional activity of PCK1. Meanwhile, treatment with 666‐15 resulted in the opposite experimental results (Supplementary Figure 8K). The promoter and 5′‐UTR region of the human PCK1 gene contains one site that matches the CREB1‐binding sequence, respectively. Besides, PCK1 knockdown partially restored the inhibition of cell proliferation caused by ZIP6 reduction (Supplementary Figure 8L,M). Taken together, the above data demonstrated that CREB1 suppressed PCK1 transcriptional activity due to its ability to directly bind to the 5′‐UTR region of PCK1.

Treatment with the inhibitors of glycolysis enzyme HK2, glutathione S‐transferase1 (GSTP1) and aspartyl‐tRNA synthetase (Asp‐AMS) had a very distinct effect on the sensitivity to knocking‐down ZIP6 or addition of 666‐15 as compared with control in a dose‐dependent manner (Figure 2E; Supplementary Figure 9A–C). Similarly, these inhibitors also caused more significant mitochondrial depolarization in the presence of 666‐15 (Supplementary Figure 9D). These results indicated mitochondrial dysfunction (depolarization) induced by silencing SLC39A6‐CREB1 axis may result in liver cancer cells are sensitive to some Metabolic inhibitors. As well, in addition to these common inhibitors, LIHC patients with low expression of ZIP6 are also more sensitive to some drugs, such as Erlotinib, TGX221 and Rapamycin (Supplementary Figure 9E). Therefore, these data provide theoretical and practical guidance for the development of drugs targeting SLC39A6.

In summary, based on our mechanistic studies, we demonstrated that SLC39A6 activated CREB1, and inhibited PCK1 expression, which promoted LIHC progression (Supplementary Figure 9F). For the first time, bioinformatics analysis in combination with energy metabolomic studies and double‐luciferase assay, as well mitochondrial membrane potential detection revealed SLC39A6‐CREB1 axis that functions in LIHC progression in vitro through regulation of mitochondrial electron transport chain activity. Importantly, as well as serving as a potential therapeutic target for liver cancer, *SLC39A6* may also be an important prognostic indicator. Thus, our findings establish SLC39A6's role in LIHC development, thereby establishing a theoretical basis for developing potential prognostic biomarkers and novel therapeutic targets. Overall, targeting SLC39A6‐CREB1 axis might be exploited for therapeutic gain for LIHC. In future research, to better understand the biological role of SLC39A6, we expect to implement knockout mice in future studies to obtain precise gene expression regulation and functional analyses in vivo.

## AUTHOR CONTRIBUTIONS

The subject design was completed by Guoqiang Zhang, Ze Yu and Haijie Ma. Bioinformatics related content was completed by Ze Yu. The experiments operation was carried out by Cheng Chen, Hongpeng Gu and Jie Wang; Haijie Ma, Yingjie Zhang, and Jinliang Dong performed statistical analysis of the data. Ze Yu and Cheng Chen wrote and revised the manuscript. All authors contributed to discussing the results and approved the final manuscript.

## FUNDING INFORMATION

This work was supported by grants from the China Postdoctoral Science Foundation (2020M680120).

## CONFLICT OF INTEREST STATEMENT

The authors declare no conflicts of interest.

## Supporting information


**Data S1.** Supporting Information.
