# Supplementary material for: Suppression of SLC39A6‐CREB1 axis in liver cancer causes PCK1‐mediated mitochondrial dysfunction
Source: Cell Prolif. 2023 Jul 12;56(10):e13527. doi: 10.1111/cpr.13527 (PMC10542647; doi:10.1111/cpr.13527)
Supplement: Supplementary file 1 — Data S1. Supporting Information. [file CPR-56-e13527-s001.docx]

**Supplementary Files**

**Supplementary Methods and Materials**

***Cell culture and viability***

The human liver cancer cell lines HepG2 and HLE were obtained from ATCC and grown in DMEM (Gibco, California, USA) supplemented with 10% fetal bovine serum (FBS, HyClone, UT, USA), 100 U/ml penicillin, and 100 μg/ml streptomycin (Gibco). All cells were carried out at 37˚C with 5% CO2 in the incubator (Thermo Fisher). Cell passaging and seeding were conducted when the cell coverage reached at 70%-80%. The CCK-8 kit (Vazyme, Nanjing, China) was used to detect cell viability.

***Intracellular metabolites measurements***

The intracellular metabolites in this study were determined by liquid chromatography-mass spectrometry. Separation was achieved using the Agilent 1290 Infinity LC ultra-high performance liquid chromatography system. The mobile phase was composed of liquid a containing 10 mM ammonium acetate aqueous solution, liquid B containing acetonitrile, and the sample was injected automatically. A 45-degree column, a flow rate of 300 liters per minute, and a volume injection of 2 liters were used. In the sample queue, a QC sample is set at a certain number of experimental samples at intervals, which is used to detect and evaluate the stability and repeatability of the system. An inductively coupled plasma mass spectrometer, model 5500 QTRAP, was used to analyze the samples in the negative ion mode. Multi-quant software was used to extract the chromatographic peak area and retention time. Metabolites were identified via adjusting the retention time by the standard of energy metabolic substances. LC-MS experiment process is completed by APTBIO (shanghai applied protein technology co.ltd).

***ROS detection***

ROS levels were measured with DCFH-DA (molecular fluorescent probes, Beyotime, China). The complete medium was removed and replaced with 5 mM DCFH-DA. The complete medium was removed after 24 hour and replaced with 5 μM DCFH-DA in medium. After 30-min incubation at 37°C, cells were washed with PBS. At last, fluorescence signal was detected using the BD Flow cytometer. Data were analyzed using FlowJo 7.6 software.

***Cell transfection***

Small interfering RNAs (siRNAs) targeting SLC39A6 were designed and purchased from Tsingke Biotechnology Co.,Ltd. (Beijing). The coding sequences of PCK1 were cloned into the pcDNA3.1(-) vector, the coding sequences of SLC39A6 were separately cloned into the pcDNA3.1(-) vector and pEGFP-N2 vector. For plasmid and siRNA transfection, Lipofectamine 3000 (L3000015, Invitrogen, USA) and Lipofectamine RNAiMAX (#13778075, Invitrogen, USA) was employed. After 48h post-transfection, the treated cells were used for subsequent experiments including assays for cell proliferation, migration, qRT-PCR, and western blotting. The sequences of siRNAs, qRT-PCR primers, antibody and inhibitors are listed in ***Supplementary Files***.

***RNA extraction and qRT-PCR analysis***

Total RNA was extracted with Trizol Reagent. Reverse transcription was performed with the HiScript® III 1st Strand cDNA Synthesis Kit (Vazyme, China). qRT-PCR was carried out with Taq Pro Universal SYBR qPCR Master Mix (Vazyme, China) to the manufacturer's procedure. β-actin was used as the internal control. Relative gene expression was calculated using the 2-ΔΔCt method.

***Luciferase reporter assay***

The effect of CREB1 on the transcription activity of PCK1 was explored using the dual-luciferase reporter assays. Sequences of PCK1 promoter containing CREB1 binding sites and their control deletion sites were cloned to the pGL4.1-Basic-luc-reporter vector. Dual-Luciferase Reporter assay system (Promega, WI, USA) was used to assess luciferase activity after the plasmids were co-transfected with slc39a6 plasmids.

***Western blotting***

Total protein lysates were prepared in RIPA buffer (Beyotime, China) with 1% protease inhibitor cocktail and phosphatase inhibitor. Protein concentration was measured by Bradford Protein assay kit (Beyotime, China). The proteins were separated and transferred using SDS-PAGE, transferred onto polyvinyllidene fluoride (PVDF) and blocked with 5% BSA for 1 h at room temperature. The membranes were incubated with the corresponding primary antibodies at 4 °C overnight, and then washed with TBST followed by incubation with HRP secondary antibodies for 1 hour and to rinsing three times in TBST. Proteins were detected and visualized using the chemiluminescent HRP substrate (Millipore, USA), and normalized to the corresponding to the β-actin level. The antibodies used in the current study are listed in ***Supplementary Table S1***.

***Measurement of mitochondrial membrane potential***

For the detection and measurement of mitochondrial membrane potential, we used the specific fluorescent probes TMRE (Beyotime, China). Cells were cultured and incubated with 5 μM TMRE for 30 min at 37˚C in the dark, according to the manufacturer's protocol. The fluorescence was immediately observed under a Leica DMI3000 B fluorescence microscope, and fluorescence levels were then measured by software Image pro plus. The main reagents and kits used in this study are displayed in ***Supplementary Table S2.***

***Antibody microarray***

Antibody microarray experiment was performed using the Proteome Profiler Human Phospho -Kinase Array Kit (ARY003C, R&D systems, USA) in accordance with the instruction.

***Statistical analysis***

All data were analyzed using the Prism 8.0 (GraphPad Software Incorporated, USA). The significance of differences was examined by Student's t-test. The results represent the mean ± SD from at least three independent experiments. P < 0.05 was considered statistically significant.

***Bioinformatics analyses***

- 1. RNA-sequencing expression (level 3) profiles and corresponding clinical information for LIHC were downloaded from the TCGA dataset. Consistency analysis by using Consensus Cluster Plus R package (v1.54.0), the maximum number of clusters is 6, and 80% of the total sample is drawn 100 times, clusterAlg="hc", innerLinkage='ward.D2'. Use the R software (v1.0.12) for clustering heatmaps. The gene expression heatmap retains genes with SD > 0.1. If the number of input genes is more than 1000, it will extract the top 25% genes after sorting the SD. R package were implemented by R version 4.0.3.
  2. RNA-sequencing expression profiles and corresponding clinical information for LIHC were downloaded from the TCGA dataset. Using the lim-ma package in the R software to study the differentially expressed mRNA. “Adjusted P < 0.05 and Log2 (Fold Change) >1 or Log2 (Fold Change) < −1” were defined as the threshold for the differential expression of mRNAs. To further confirm the underlying function of potential targets, these data were analyzed by functional enrichment and KEGG Analysis. To better understand the carcinogenesis of mRNA, Cluster Profiler package (version: 3.18.0) in R was employed to analyze the GO function of potential targets and enrich the KEGG pathway. The R software ggplot2 package was used to draw boxplot; the R software pheat-map package was used to draw heat-map.
  3. Converting counts data to TPM and normalizing data log2 (TPM+1), keeping samples with clinical information. Finally, there are liver cancer samples for subsequent analysis. Log-rank test was used to compare differences in survival between these groups. The time-ROC (v 0.4) analysis was used to compare the predictive accuracy of SLC39A2, SLC39A6, SLC39A10 and SLC39A14 gene and risk score. **Lasso**: The least absolute shrinkage and selection operator (LASSO) regression algorithm was used for feature selection, 10-fold cross-validation was used, and the R package glmnet was used for the analysis; **Cox**: Multivariate cox regression analysis was used to construct a prognostic model, and the R package survival used for the analysis; **Step**: First, the multi -factor cox regression was used to analyze the data, and then the step function performed the iteration. **Finally**, the optimal model is selected as the final model. For Kaplan-Meier curves, p-values and hazard ratio (HR) with 95% confidence interval (CI) were generated by log-rank tests and univariate cox proportional hazards regression.
  4. The HCC transcriptomic data analyzed in the study were downloaded from Gene Expression Omnibus (GEO) database (number **GSE136247** and **GSE99807**).
  5. The gene correlation analysis data come from ENCORI.

<http://starbase.sysu.edu.cn/panCancer.php>

- 1. The gene regulatory network was constructed by Gene-MANIA. https://genemania.org/
  2. Expression of SLC39A6 in LIHC based on individual cancer stages and nodal metastasis status were analyzed using data from the ualcan. <http://ualcan.path.uab.edu/>
  3. The survival curves were analyzed using data from the GEPIA website.

http://gepia.cancer-pku.cn/

- 1. The ROC analysis data is from UCSC database. https://xenabrowser.net/
  2. The correlations between individual gene and pathway score was analyzed with Spearman.
  3. Heat-map of negatively correlated significant genes with ZIP6 is derived from LinkedOmics.

<http://www.linkedomics.org/login.php>

- 1. RNA-sequencing expression profiles and corresponding clinical information for LIHC were downloaded from the TCGA dataset. Two-gene correlation map is realized by the R software package ggstatsplot, and the multi-gene correlation pheatmap is displayed by the R software package. Spearman's correlation analysis to describe the correlation between quantitative variables without a normal distribution. P values less than 0.05 were considered statistically significant (*P < 0.05).
  2. The gene correlation analysis data (heatmap) come from UCSC Xena. https://xena.ucsc.edu/
  3. We obtained the raw data of single-cell transcriptome profiling from the Gene Expression Omnibus (GEO) database (**GSE3064818**). Then, we utilized the Seurat package to generate the object, filtered out cells with poor quality. We conducted standard data preprocessing, where we calculated the percentage of the gene numbers, cell counts and mitochondria sequencing count. We excluded genes with less than only 3 cells detected and disregarded cells with less than 200 detected gene numbers.
  4. The resource of genome-wide CRISPR-Cas9 knockout screens in hundreds of cancer cell lines. http://[www.depmap.org](http://www.depmap.org)
  5. The Comprehensive Database for Regulations of Human Transcription Factors: hTFtarget. http://bioinfo.life.hust.edu.cn/hTFtarget/
  6. The database of eukaryotic transcription factors: TRANSFAC. <https://genexplain.com/transfac/>
  7. The database of transcription factor binding profiles: JASPAR. https:// jaspar.genereg.net/
  8. Drug susceptibility score (IC50 assesses drug efficacy): RNA-sequencing expression profiles and corresponding clinical information for LIHC were downloaded from the TCGA dataset. Predicted the chemotherapeutic response for each sample based on the largest publicly available pharmacogenomics database the Genomics of Drug Sensitivity in Cancer (GDSC). The prediction process was implemented by R package “pRRophetic”. The samples' IC50 was estimated by ridge regression. All parameters were set as the default values. Using the the batch effect of combat and tissue type of all tissues, and the duplicate gene expression was summarized as mean value. The above analysis methods and R package were implemented by R foundation for statistical computing version 4.0.3.
  9. HCCDB: a database of hepatocellular carcinoma expression atlas.

<http://lifeome.net/database/hccdb/home.html>

***RNA interference and qRT-PCR primers***

**siRNA sequences**: Design of siRNA: siDirect version 2.0 (<http://sidirect2.rnai.jp/>)

SLC39A6:

Si-#1: 5’- CCAACACGUGGUGGAAUUUTT -3’; 5’- AAAUUCCACCACGUGUUGGTT -3’

Si-#2: 5’- GCAUAGCAGCUAAGAUCUUTT -3’; 5’- AAGAUCUUAGCUGCUAUGCTT -3’

PCK1:

Si-PCK1.1: 5’- GCUGAAGAAGUAUGACAACTT -3’; 5’- GUUGUCAUACUUCUUCAGCTT -3’

Si-PCK1.2: 5’- CAAGACGGUUAUCGUCACCTT -3’; 5’- GGUGACGAUAACCGUCUUGTT -3’

**Primer sequences (qRT-PCR dtection)**:

1. SLC39A6 (F1/R1)

Up: 5’- ATGGCCTAGCAATTGGTGCT-3’; Down: 5’- AACGGTCATGCCAGCCTTTA-3’

1. COX17

Up: 5’- TGCGTGTATCATCGAGAAAGGA-3’; Down: 5’- GCCTCAATTAGATGTCCACAGTG-3’

1. NDUFS3

Up: 5’- TGTGGCTGAAATCTTGCCCAA’; Down: 5’- AGTTGAAGCGCAGAGACAACA-3’

1. NDUFA1

Up: 5’- GCGTACATCCACAGGTTCACT-3’; Down: 5’- GCGCCTATCTCTTTCCATCAGA-3’

1. NDUFB1

Up: 5’- GTCCCTATGGGATTTGTCATTGG-3’; Down: 5’- CAGTTAGCCGTTCATCACTCTT-3’

1. TOMM20

Up: 5’- GGTACTGCATCTACTTCGACCG’; Down: 5’- TGGTCTACGCCCTTCTCATATTC-3’

1. COX5A

Up: 5’- GCCAGATATAGATGCCTGGGA-3’; Down: 5’- ACAACCTCTAGGATACGAACTGT-3’

1. ATP5B

Up: 5’- CCTGTCAGGGACTATGCGG-3’; Down: 5’- TCCTTACTGTGCTCTCACCCA-3’

1. PCK1

Up: 5’- TTGAGAAAGCGTTCAATGCCA-3’; Down: 5’- CACGTAGGGTGAATCCGTCAG-3’

1. SLC39A6 (F2/R2)

Up: 5’- TCCTAGAAACAGCCAGGGGA-3’; Down: 5’- ACCCGGCTCTTTGATGTGAC-3’

1. CCND1

Up: 5’- GCTGCGAAGTGGAAACCATC-3’; Down: 5’- CCTCCTTCTGCACACATTTGAA-3’

1. CCNE1

Up: 5’- ACTCAACGTGCAAGCCTCG’; Down: 5’- GCTCAAGAAAGTGCTGATCCC-3’

1. CDC6

Up: 5’- CCAGGCACAGGCTACAATCAG-3’; Down: 5’- AACAGGTTACGGTTTGGACATT-3’

1. ABL1

Up: 5’- CATCACGCCAGTCAACAGTCT-3’; Down: 5’- GTACACCCTCCCTTCGTATCT-3’

1. CCNA2

Up: 5’- CGCTGGCGGTACTGAAGTC-3’; Down: 5’- GAGGAACGGTGACATGCTCAT-3’

1. CCNB1

Up: 5’- AATAAGGCGAAGATCAACATGGC-3’; Down: 5’- TTTGTTACCAATGTCCCCAAGAG-3’

S

**Primer sequences (plasmid construction)**:

1. ZIP6-O: Vector: pcDNA3.1(-): *Xho1/BamH1*

Up: 5’- CCG*CTCGAG*ATGGCGAGGAAGTTATCTGTAAT-3’; *Xho1*

Down: 5’- CGC*GGATCC*CTAGAAATTTATACGAAACACG-3’ *BamH1*

1. PCK1-Promoter-Luc(L1): Vector: pGL3-basic: *Sma1/HindⅢ*

Up: 5’- TCC*CCCGGG*TCTAGCAAAACTGCCAAGCCC-3’; *Sma1*

Down: 5’- CCC*AAGCTT* TTCTGCAGAGTGCTGCTAAGG-3’ *HindⅢ*

1. PCK1-Promoter-Luc(L2): Vector: pGL3-basic: *Sma1/HindⅢ*

Up: 5’- TCC*CCCGGG* GCCTTCCCACTGGGAACACAA -3’; *Sma1*

Down: 5’- CCC*AAGCTT* TTCTGCAGAGTGCTGCTAAGG -3’ *HindⅢ*

1. PCK1-Promoter-Luc(P1): Vector: pGL3-basic: *Sma1/HindⅢ*

Up: 5’- TCC*CCCGGG*TCTAGCAAAACTGCCAAGCCC -3’; *Sma1*

Mut-down: GGAGGCTCGCCACGGGGGCAGGTCTCT

Mut-up: AGAGACCTGCCCCCGTGGCGAGCCTCC

Down: 5’- CCC*AAGCTT* TTCTGCAGAGTGCTGCTAAGG -3’ *HindⅢ*

1. PCK1-Promoter-Luc(P2): Vector: pGL3-basic: *Sma1/HindⅢ*

Up: 5’- TCC*CCCGGG*TCTAGCAAAACTGCCAAGCCC -3’; *Sma1*

Mut-down: AAGGGGCACGAATGCCTTTCTTCTCTT

Mut-up: AAGAGAAGAAAGGCATTCGTGCCCCTT

Down: 5’- CCC*AAGCTT* TTCTGCAGAGTGCTGCTAAGG -3’ *Hind*

**Supplementary Figures and figure legends**

**
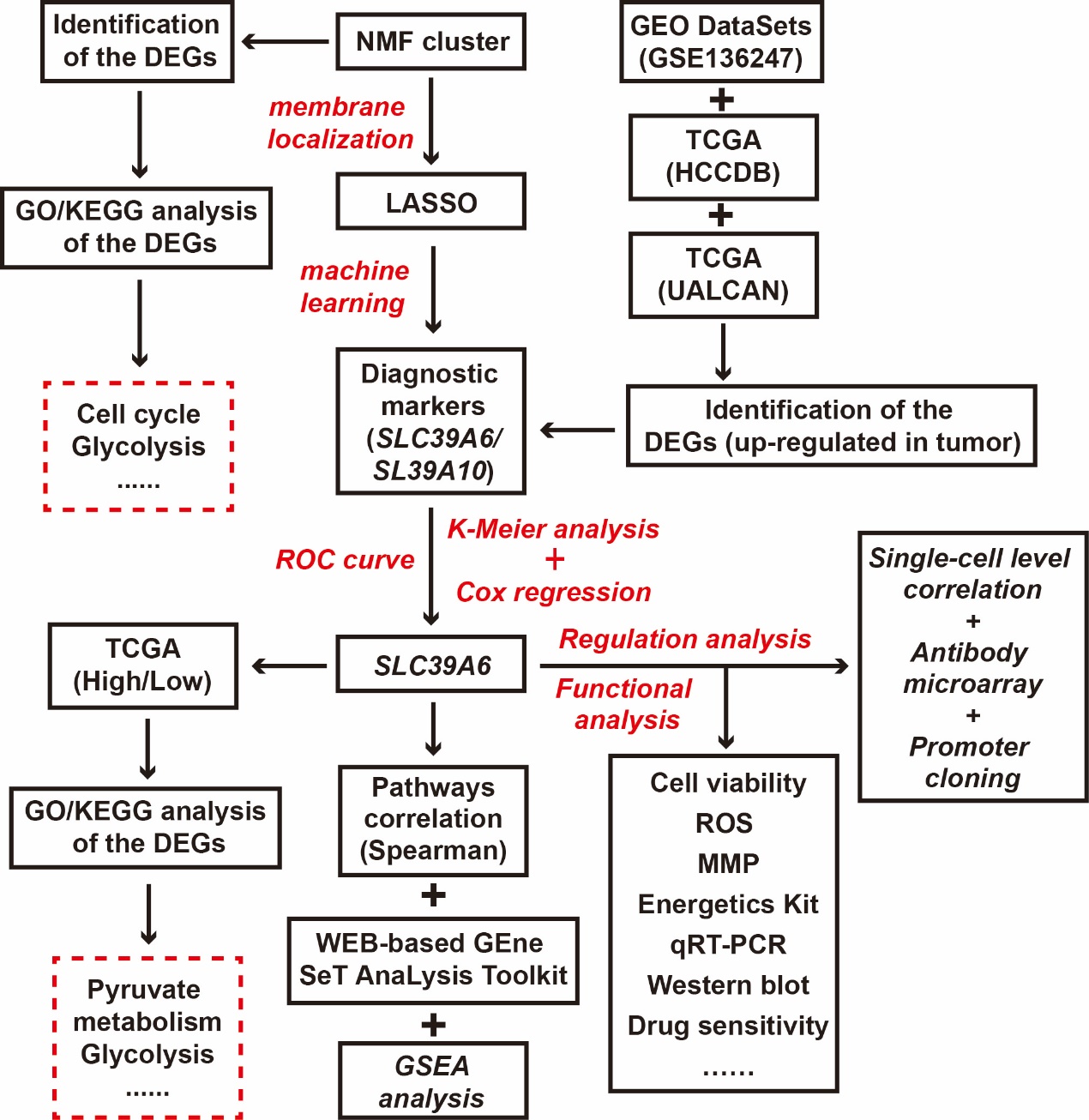
**

**Supplementary Figure 1. The flowchart depicting the investigation procedure.**

**
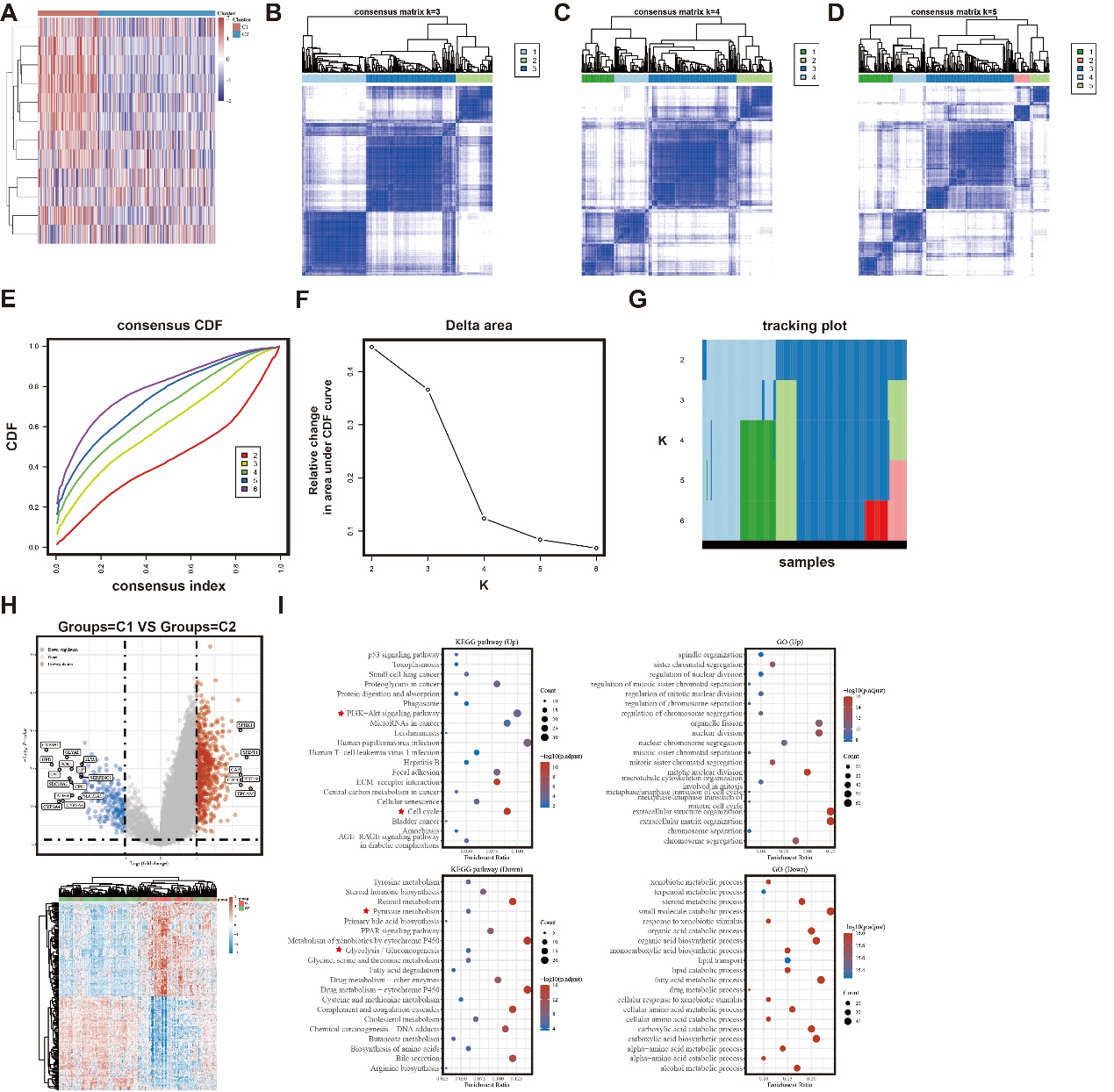
**

**Supplementary Figure 2. Identification of subclasses identification based on 14 SLC39A-related genes using NMF consensus. (A)** Consensus matrix heatmap for k=2. **(B-D)** Consensus matrixes for k=3 to k=5 of the 371 LIHC patients in the TCGA datasets by clustering the gene expression profile of the 14 SLC39A-related genes. **(E)** CDF value of consensus index. **(F)** Relative change in area under CDF curve for k=2-6. **(G)** The tracking plot for k=2-6. **(H)** Volcano plot of the DEGs in cluster 1 and cluster 2. **(I)** The bubble plot of GO and KEGG pathway enriched with DEGs.

**
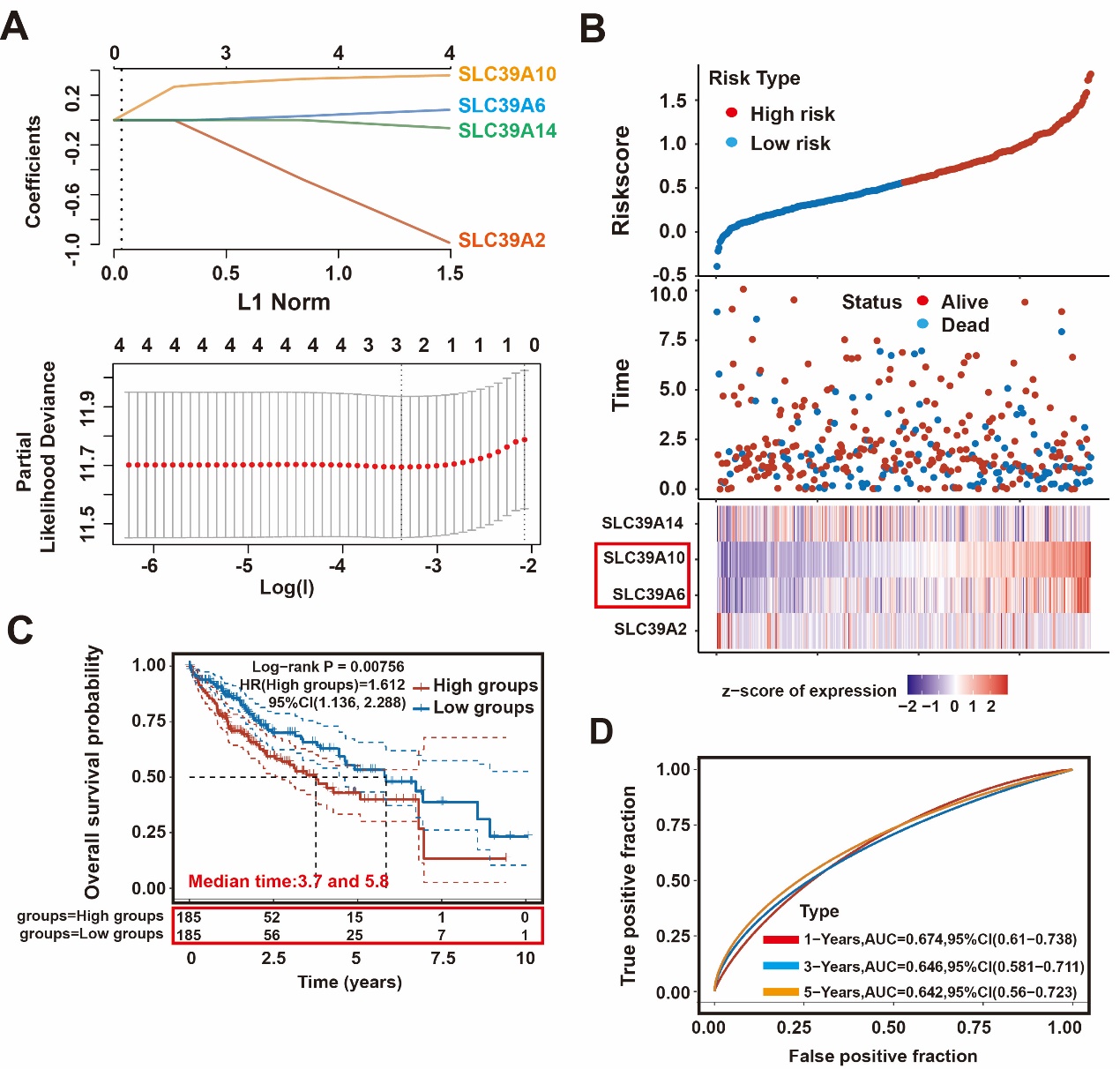
**

**Supplementary Figure 3. Risk score analysis based on 4 SLC39A genes signature in LIHC patients. (A)** Ten-fold cross-validation for the coefficients of four SLC39A genes in the LASSO model. **(B)** Distributions of the risk scores, survival status, and expression of the four SLC39A genes, in patients with LIHC. **(C)** KM analysis of overall survival of patients with LIHC, based on the four SLC39A. **(D)** ROC curve analysis of the four SLC39A genes for 1, 3, and 5 years.

**
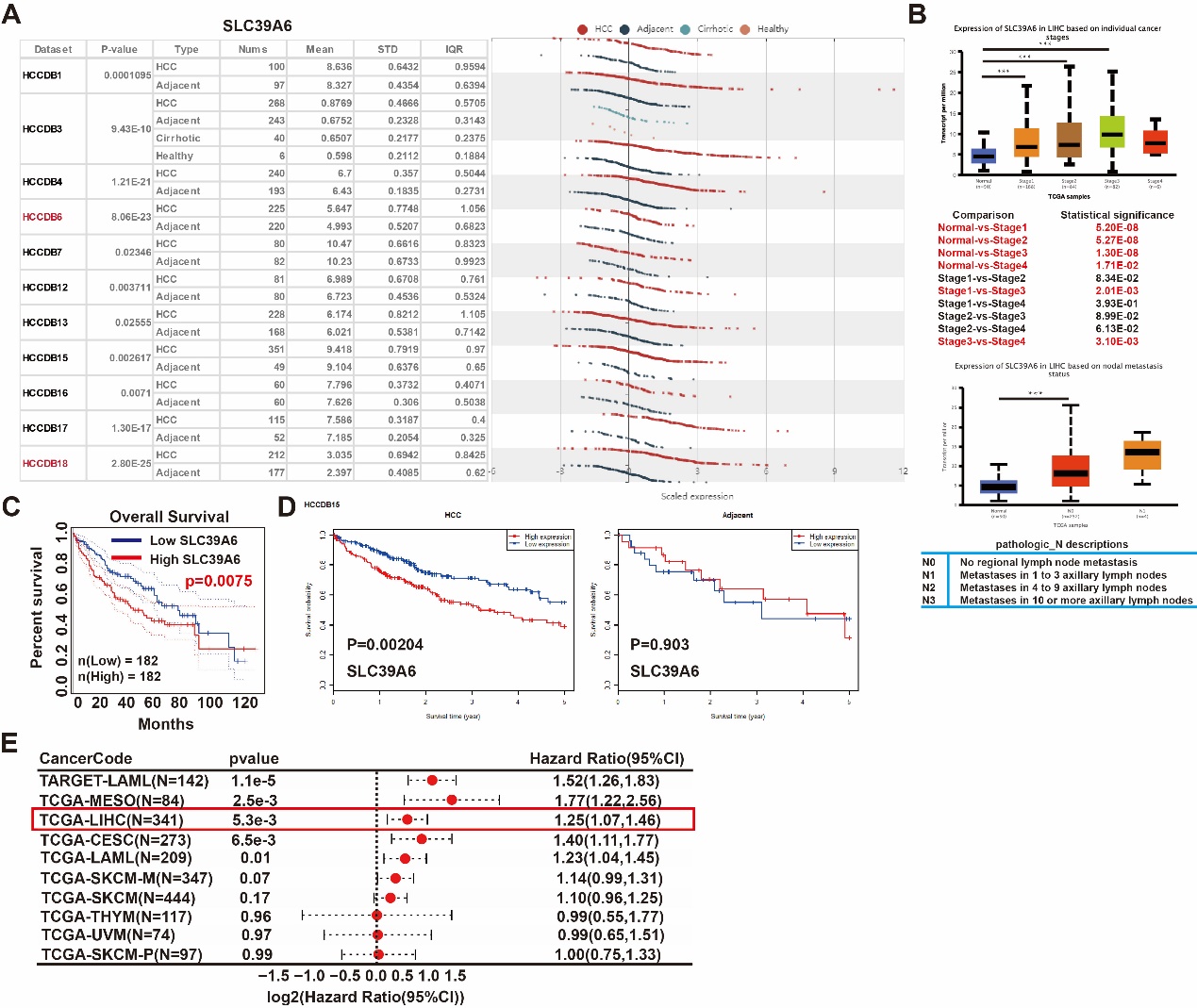
**

**Supplementary Figure 4. SLC39A6 expression was increased in LIHC (A)** SLC39A6 expression levels were obtained from the HCCDB website. **(B)** Expression of SLC39A6 in LIHC based on individual cancer stages and nodal metastasis status. **(C, D)** High SLC39A6 expression related to poor prognosis of LIHC. **(E)** Forest plot shows the prognosis of SLC39A6 in ten tumors.

**
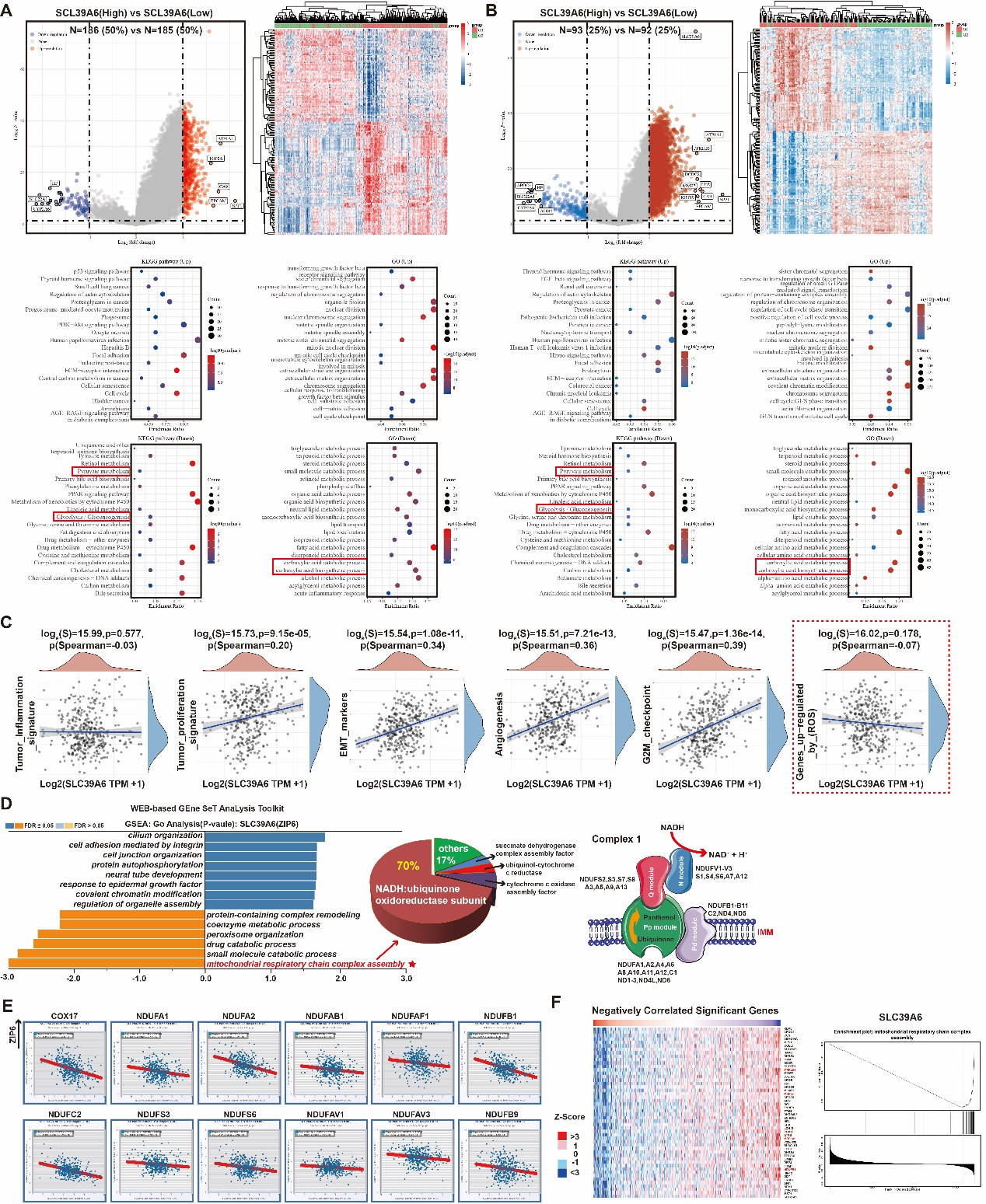
**

**Supplementary Figure 5. Biological pathway analysis of the SLC39A6-related genes in LIHC. (A, B)** The volcano map showed the differential genes between patients with high SLC39A6 levels (50%) vs. low SLC39A6 levels (50%), and high SLC39A6 levels (25%) vs. low SLC39A6 levels (25%), respectively. GO and KEGG enrichment analysis for these DEGs. **(C)** The relationship between SLC39A6 and six tumorigenic pathways in LIHC. **(D)** GO-Biological process enrichment analysis for these genes of SLC39A6 negatively correlated. **(E)** The correlation analysis between SLC39A6 and NADH ubiquinone oxidoreductase subunit. **(F)** Enrichment plots of genes negatively correlated with SLC39A6 by performing GSEA.


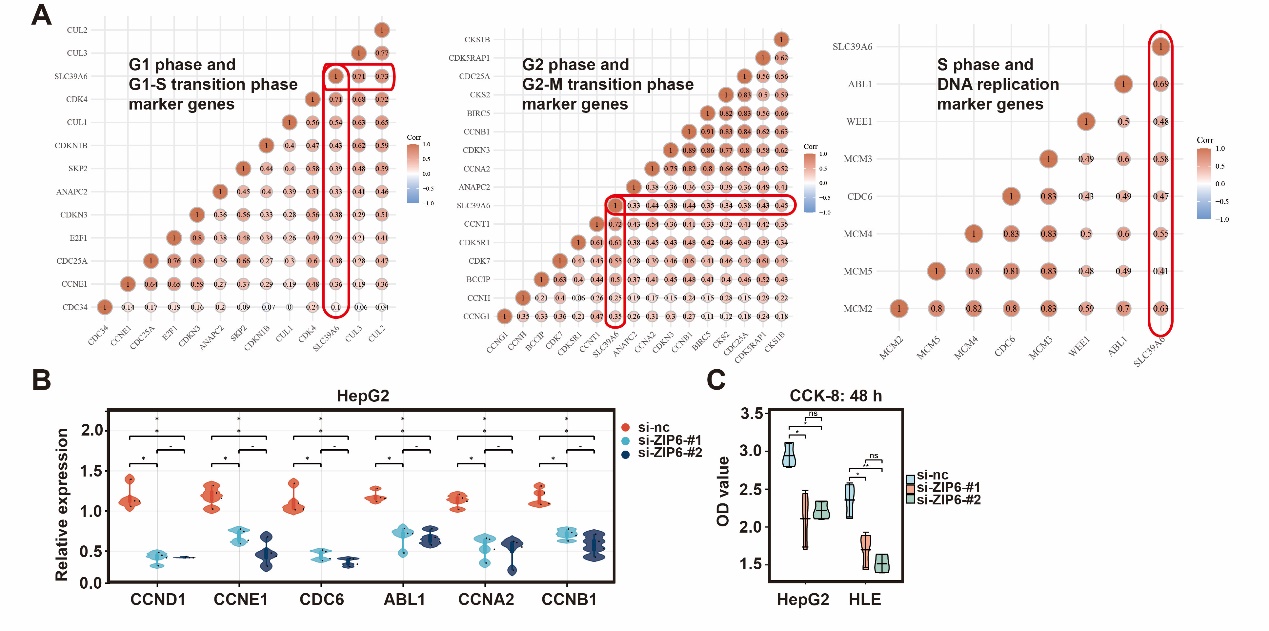


**Supplementary Figure 6. SLC39A6 enhanced the proliferation of lIHC cells. (A)** The correlations between cell cycle marker genes and SLC39A6. **(B)** qPCR of cell cycle-related genes. **(C)** The proliferative ability of HLE cells was detected by CCK-8 assays. *P< 0.05, **P< 0.01.

**
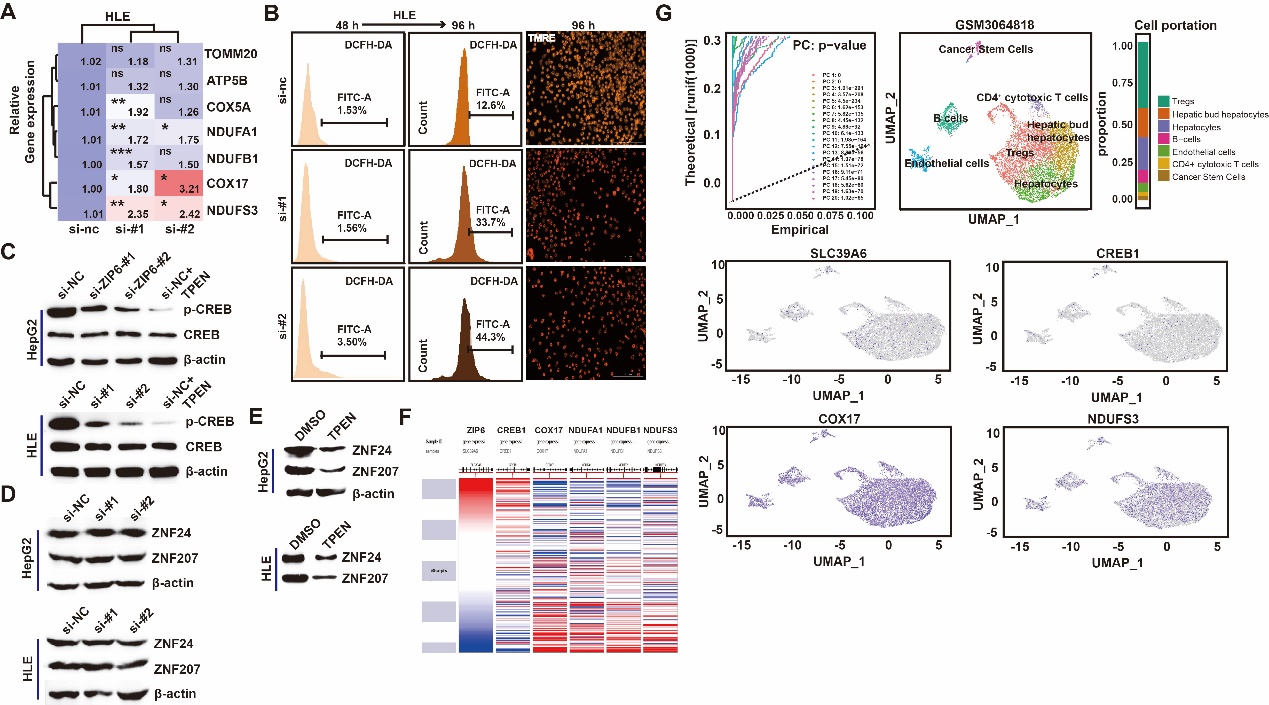
**

**Supplementary Figure 7. SLC39A6 facilitates metabolic reprogramming of LIHC cells. (A)** qPCR was used to evaluate the electron transport chain related genes expression levels in SLC39A6 knockdown cells. **(B)** ROS detection and MMP assay for HLE cells treated with si-SLC39A6 mimics. **(C)** CREB phosphorylation was downregulated in LIHC cells treated with TPEN and knocked-down SLC39A6. **(D, E)** Western blot assay for ZNF24 and ZNF207 in LIHC cells treated with TPEN and SLC39A6 knock-down. **(F)** mRNA expression levels of ZIP6, CREB1, COX17, NDUFA1, NDUFB1 and NDUFS3 in liver cancer samples as plotted on a heat-map. **(G)** Correlation of SLC39A6, CREB1, COX17 and NDUFS1 via the single cell analysis of data (GSE3064818).

**
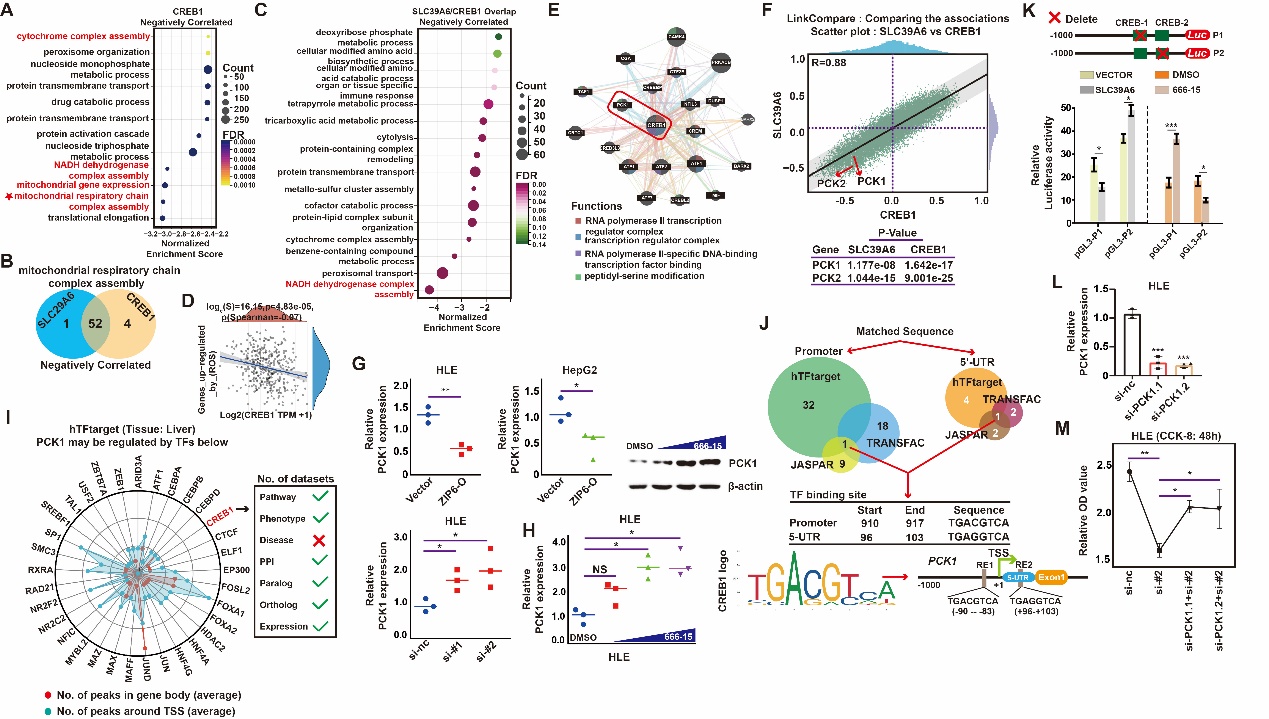
**

**Supplementary Figure 8. SLC39A6-CREB1 axis is negatively correlated with PCK1. (A)** Top 12 enriched GO terms for genes negatively correlated with CREB1. **(B)** Overlap of the genes negatively correlated with CREB1 and SLC39A6. **(C)** The top 16 enriched GO terms for genes negatively correlated with both CREB1 and SLC39A6. **(D)** The relationship between CREB1 and genes up-regulated by ROS in LIHC. **(E)** Gene-MANIA constructed a protein-protein interaction network involving in CREB1. **(F)** The correlation of PCK1 and PCK2 genes with CREB1 and SLC39A6 via LinkCompare analysis. **(G)** qRT-PCR detected relative PCK1 expression in cells transfected with si-ZIP6 or ZIP6-overexpressed vector. **(H)** qRT-PCR and WB detected relative PCK1 expression in cells treated with different concentration of 666-15. **(I)** Analysis of predicted proteins that regulated PCK1 transcription via hTFtarget. **(J)** Schematic diagram of CREB1 binding location and sequence on PCK1 gene. **(K)** Dual-luciferase reporter assays were performed in HLE cells to confirm the exact CREB1 binding region on the PCK1 promoter. **(L)** qRT-PCR detected relative PCK1 expression in HLE cell transfected with siRNAs of PCK1. **(M)** Knockdown of PCK1 on the proliferation of HLE cells interfered with ZIP6. *P< 0.05, **P< 0.01.


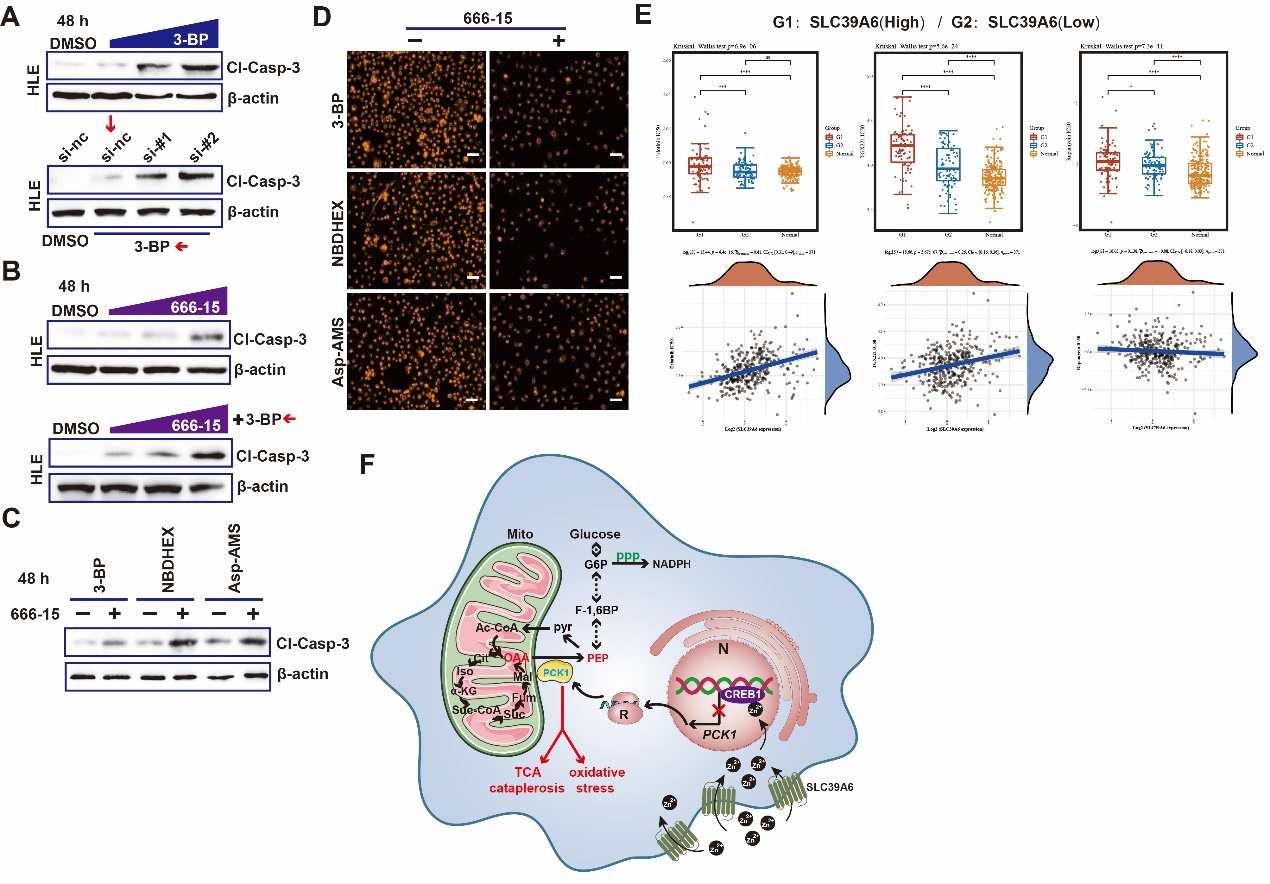


**Supplementary Figure 9. (A)** WB analysis of Cl-Caspase-3 in cells treated with low concentration of 3-BP combined with SLC39A6 knock-down. **(B)** WB analysis of Cl-Casp-3 in HLE cells treated with different doses of 666-15 combined with 3-BP. **(C)** WB analysis of Cl-Caspase-3 in cells treated with 3 metabolic inhibitors combined with 666-15. **(D)** MMP analysis in HLE cells treated with 3 metabolic inhibitors combined with 666-15. **(E)** Spearman correlation analysis between IC50 scores for three clinical drugs and SLC39A6 gene expression. **(F)** A model of the regulatory mechanism of SLC39A6-CREB1-PCK1 axis in LIHC.

**Table S1. Primary antibodies used in this study**

| **Antigens** | **Manufacturer** | **Catalog Number** | **Application** |  |
| --- | --- | --- | --- | --- |
| ZIP6 (LIV-1) | Proteintech | 14236-1-AP | 1:500 for WB |  |
| COX17 | Proteintech | 11464-1-AP | 1:1000 for WB |  |
| NDUFS3 | Proteintech | 15066-1-AP | 1:1000 for WB |  |
| beta-actin | ABclonal | AC026 | 1:3000 for WB |  |
| Phospho-CREB1 | CST | # 9198S | 1:1000 for WB |  |
| CREB1 | CST | # 9197S | 1:1000 for WB |  |
| ZNF24 | Proteintech | 11219-1-AP | 1:1000 for WB |  |
| ZNF207 | Proteintech | 14814-1-AP | 1:1000 for WB |  |
| PCK1 | Proteintech | 16754-1-AP | 1:1000 for WB |  |
| Cleaved-Caspase-3 | ABclonal | A11021 | 1:500 for WB |  |

**Table S2. Kits and reagents used in this study**

| **Kits/Reagents** | **Manufacturer** | **Catalog Number** | **Application** |  |
| --- | --- | --- | --- | --- |
| TRIzol | Invitrogen | 15596026 | RNA extraction |  |
| HiScript III* | Vazyme | R312-02 | RT | |
| SYBR qPCR Mix | Vazyme | Q712-02 | Q-pcr |  |
| Lipofectamine 3000 | Invitrogen | L3000015 | Transfection |  |
| Lipofectamine RNAiMAX | Invitrogen | 13778075 | Transfection |  |
| Apoptosis Detection | Vazyme | A211-01 | Apoptosis |  |
| DCFH-DA | Beyotime | S0033S | ROS Assay Kit |  |
| CCK-8 Cell count kit | Vazyme | A311-01 | CCK8 assay |  |
| TMRE | Beyotime | C2001S | MMP detection |  |
| Dual Luciferase | Vazyme | DL101-01 | Dual Luciferase |  |
| Reporter Assay Kit |  |  |  |  |
| 3-Bromopyruvic acid(3-BP) | MCE | HY-19992 | inhibitor |  |
| PKM2-IN-1 | MCE | HY-103617 | inhibitor |  |
| TEPP-46 | MCE | HY-18657 | inhibitor |  |
| FX-11 | MCE | HY-16214 | inhibitor |  |
| GSK2837808A | MCE | HY-100681 | inhibitor |  |
| NBDHEX | MCE | HY-135318 | inhibitor |  |
| Asp-AMS | MCE | HY-112860 | inhibitor |  |
| MSDC-0602 | MCE | HY-108022 | inhibitor |  |
| UK-5099 | MCE | HY-15475 | inhibitor |  |
| CCI-006 | MCE | HY-114410 | inhibitor |  |

**HiScript III***: HiScript® III 1st Strand cDNA Synthesis Kit (+gDNA wiper); **SYBR qPCR Mix**: Taq Pro Universal SYBR qPCR Master Mix; **Apoptosis Detection**: Annexin V-FITC/PI Apoptosis Kit.

**Abbreviations**

CREB1: cAMP responsive element binding protein 1; DARS1: aspartyl-tRNA synthetase 1; GEO: Gene Expression Omnibus; GO: Gene Ontology; GSTP1: Glutathione S-transferase pi 1; HK2: hexokinase-2; LIHC: Hepatocellular carcinoma; MMP: mitochondrial membrane potential; MPC: Mitochondrial pyruvate carrier; OS: Overall survival; OXPHOS: oxidative phosphorylation; PCK1: phosphoenolpyruvate carboxykinase 1; PC: pyruvate carboxylase; PEP: phosphoenolpyruvate; PKM: pyruvate kinase M1/2; qRT-PCR: Quantitative Real‑time PCR; ROS: Reactive oxygen species; SLC39A6: solute carrier family 39 member 6; TCA: tricarboxylic acid; TCGA: The Cancer Genome Atlas; TPEN: N,N,N0,N tetrakis (2-pyridylmethyl) ethylenediamine; WB: Western blot. NDUFS3: NADH: ubiquinone oxidoreductase core subunit S3; LDHA: lactate dehydrogenase A; COX17: cytochrome c oxidase copper chaperone COX17.
